# Development of Graded Transparent Soft Clay and Its Application in Visualization Tests of Soft Soil Foundations

**DOI:** 10.3390/ma19173747

**Published:** 2026-09-03

**Authors:** Mingyuan Wang, Jianxin He, Hesheng Cheng, Jinhua Ding

**Affiliations:** 1 PowerChina Huadong Engineering Corporation Limited, Hangzhou 311100, China; wang_my2@hdec.com; 2 College of Hydraulic and Civil Engineering, Xinjiang Agricultural University, Urumqi 830052, China; 13475389680@163.com (H.C.); dingjh@xjau.edu.cn (J.D.); 3 Technical Research Center of Xinjiang Hydro-Geotechnical and Structural Engineering, Urumqi 830052, China

**Keywords:** graded transparent soil, soft clay, mechanical properties, visual model test, geotechnical visualization

## Abstract

Looking at the demand for precise matching between the mechanical properties of similar materials and natural soft clay in soft ground visual model tests, this paper proposes a preparation method for graded transparent soil based on optical testing principles and geotechnical similarity theory. Using n-dodecane and 15# white oil as pore fluids, combined with nano-fumed silica and optimally graded fused quartz sand, a graded transparent soil material system with accurately matched refractive indices was constructed. The synergistic effect of nano-fumed silica and graded quartz sand enables dual optimization of material transparency and mechanical performance. The feasibility of simulating soft ground was verified through load-plate tests, jacked pile penetration model tests, and consolidated-drained (CD) triaxial tests. The consolidation compression and direct shear test results show that the material has a compression coefficient of 1.43 MPa^−1^, a compression modulus of 1.72 MPa, a total internal friction angle φ ranging from 16.53° to 23.21°, and a total cohesion c ranging from 5.42 to 11.34 kPa. Further CD triaxial test results verified that the developed transparent soil exhibits effective shear strength parameters (*c*′ = 9.65 kPa, *φ*′ = 16.01°), which are highly consistent with the effective strength indices of natural Shaoxing silty clay (*c*′ = 12.94 kPa, *φ*′ = 13.52°), with a relative error of ultimate deviator stress less than 5% under various confining pressures. Moreover, different strength levels can be obtained by adjusting the pre-consolidation pressure. During shear loading, the material presents stable volumetric contraction without dilatancy, conforming to the typical contractive mechanical characteristics of normally consolidated soft clay. PIV velocity-field analysis of the indoor plate load test under constant-settlement-rate conditions revealed that the transparent soil displays a typical Prandtl failure mode, consistent with the failure characteristics of natural soft clay. The model test of jacked-pile penetration further confirmed that this material can effectively simulate the stress-deformation behavior of soft ground under the test conditions adopted in this study. The developed material offers a viable alternative simulant and relevant test reference for laboratory visual model investigations into soft ground deformation, pile–soil interaction and failure evolution in geotechnical model tests.

## 1. Introduction

Soft soil strata widely exist in geotechnical engineering and frequently induce foundation settlement and progressive failure under external loads. The deformation characteristics and soil–structure interaction mechanisms of soft soil foundations are essential for the safety evaluation and performance analysis of geotechnical infrastructures. Traditional geotechnical model tests are limited by the opacity of soil itself, making it difficult to directly observe the internal displacement field, initiation and propagation of shear bands, and the complete failure process within the foundation. This leads to limitations in understanding the disaster mechanisms of soft soil foundations [1]. Transparent soil technology, which uses optically transparent synthetic materials to simulate the mechanical behavior of natural soils, enables the non-invasive visual observation of internal deformation in model tests, providing a novel approach to address the above challenges [2,3].

However, the development of transparent soft clay materials has long faced the challenge of balancing transparency and mechanical properties [4]. In early research, transparent sandy soils were mostly simulated by combining amorphous silica gel with pore fluids of a matching refractive index (such as colorless mineral oils and n-alkane solvents) [5], but their lack of cohesion made it difficult to characterize the visco-elastoplastic behavior of soft clay [6]. To simulate cohesive soils, Iskander et al. [7] attempted to use amorphous silica powder with pore fluids, yet transparency in model tests was hard to guarantee. Although the fused quartz–pore fluid system achieved good optical transmission, its lack of cohesion prevented it from meeting the basic requirements for simulating soft clay [8].

Recent studies have further demonstrated that reliable experimental characterization of geomaterials is essential for understanding coupled material behavior and improving predictive capabilities in geotechnical engineering applications, highlighting the growing importance of high-quality laboratory datasets for model validation [9,10]. After over 20 years of iterative material optimization since the early 2000s, covering three successive generations of transparent soft clay formulations, differentiated material systems for transparent cohesive soil have gradually taken shape: the laponite–aqueous solution system provided a new approach for simulating natural soft clay layers [11]; amorphous silica-powder-based cohesive soil systems demonstrated good engineering applicability in model tests [12]; the U10-NaOH composite ratio (1:0.4:98.6) successfully reproduced the strength evolution and compression response characteristics of natural silt [13]. Notably, Zhou et al. [14] developed a new transparent soil based on spherical silica powder and conducted mechanical tests, showing its applicability for model tests on soil compaction effects during pile penetration [15]. Zhong et al. proposed a preparation method for transparent soil–rock mixtures using n-dodecane and white oil as the fluid phase, amorphous silica powder as the soil phase, and quartz glass particles as the rock phase, demonstrating its utility in the visualization of SRM model tests [16]. A summary of these typical transparent-soil materials and their corresponding applications is presented in Table 1.

Nevertheless, existing transparent soft clay material systems still face significant technical bottlenecks [17,18]. The primary issue is the insufficient mechanical performance of transparent soft clay prepared from single solid powders, with compressive and shear strength parameters significantly lower than those of natural soft clay prototypes [19]. Secondly, the optical properties of such materials exhibit spatial limitations, with an effective visible penetration distance typically limited to about 5 cm, which severely constrains the geometric dimensions of model tests [20].

Nano-silica particles can improve the shear strength and permeability of transparent soil by increasing the viscosity of the mixture to simulate the plasticity of natural clay and forming a spatial network structure via van der Waals forces to enhance inter-particle cohesion [21,22,23,24,25]. On this basis, graded fused quartz sand was adopted as the skeleton material to optimize the internal pore structure and simulate the irregular mineral morphology of natural soil. Different from conventional transparent sand that relies on particle mechanical interlocking to control mechanical behaviors, the macroscopic mechanical properties, shear contraction characteristics and overall mechanical responses of the developed transparent soil are primarily dominated by the nano-silica colloidal cementation system. The mechanical occlusion and interlocking effect of quartz particles is substantially weakened and no longer governs the material mechanical performance. However, the optimization of material ratios and their practical application still require experimental validation [26,27].

To address these deficiencies, this paper develops a new graded transparent soil material based on the principle of optical matching and geotechnical similarity theory. Using a blended pore fluid of n-dodecane and 15# white oil, combined with nano-fumed silica and optimally graded fused quartz sand, precise refractive index matching and enhanced mechanical performance were achieved. Systematic testing of the material’s optical properties and physico-mechanical parameters was conducted, and its engineering applicability was validated through plate load tests and jacked-pile-penetration model experiments. The research outcomes may provide a novel simulant material and technical support for visualizing the deformation mechanisms of soft roadbed foundations, pile–soil interactions, and the evolution of foundation failure in model tests.

## 2. Selection and Preparation of Materials

### 2.1. Material Characteristics

The synthesis of transparent soil materials necessitates the synergistic optimization of both optical transparency and mechanical similarity. Based on key factors such as material applicability and repeatability, this study developed a composite system comprising fused quartz sand as the soil skeleton, nano-fumed silica as the gelling medium, and a blended fluid of n-dodecane and 15# white oil to simulate the pore fluid “water”. The raw materials and their fundamental characteristic parameters are listed in Table 2.

The nano-fumed silica used was WACKER hydrophilic fumed silica N20ST, which appears as a fluffy white powder with a high specific surface area, a particle size of <50 nm, a tamped density of 40 g/L, and a tendency to adsorb volatile substances. The fused quartz sand was produced by Wanhe Mining Company (China). It features an angular, crystalline, and transparent granular morphology, with a Young’s modulus of 72 GPa.

The original available particle size range of fused quartz sand covers ultra-fine powder finer than 0.075 mm, conventional medium particle fractions of 0.075–3 mm, and oversized coarse particles of 3–5 mm. In the preliminary material optimization tests, both the ultra-fine fraction (<0.075 mm) and the oversized coarse fraction (3–5 mm) were completely excluded. The 3–5 mm oversized particles were eliminated because they caused severe light scattering, reduced overall specimen transparency, and introduced excessive internal friction. In contrast, ultra-fine quartz powder finer than 0.075 mm was discarded to avoid uneven pore-oil distribution and the attenuation of capillary cohesion provided by nano-sized fumed silica. Finally, only the qualified intermediate particle size fractions ranging from 0.075 mm to 3 mm were adopted, including six graded intervals of 0.075–0.1 mm, 0.1–0.2 mm, 0.2–0.5 mm, 0.5–1 mm, 1–2 mm, and 2–3 mm for specimen preparation. It is worth clarifying that this study aimed to achieve mechanical and deformation performance similarity with natural soft clay, rather than pursuing particle size gradation similarity.

### 2.2. Pore Fluid Preparation Methodology

The systematic refractive index characteristics of the oil-phase system, composed of n-dodecane and #15 white mineral oil, were investigated using an Abbe refractometer (ISO 6320 standard) [28]. The experimental design encompassed two research dimensions: (1) temperature dependence of refractive indices for individual mineral oils; (2) refractive index variations of mixed pore fluids (n-dodecane/#15 white mineral oil volume ratios ranging from 1:1 to 1:5) under different temperature and volumetric proportion conditions. A temperature gradient spanning 5–30 °C was established using laboratory constant-temperature air conditioning. Comprehensive measurements were conducted to determine the refractive index parameters of both mineral oils across this thermal range, followed by detailed characterization of refractive response patterns in mixed pore fluids with varying volume ratios.

**Figure 1:**
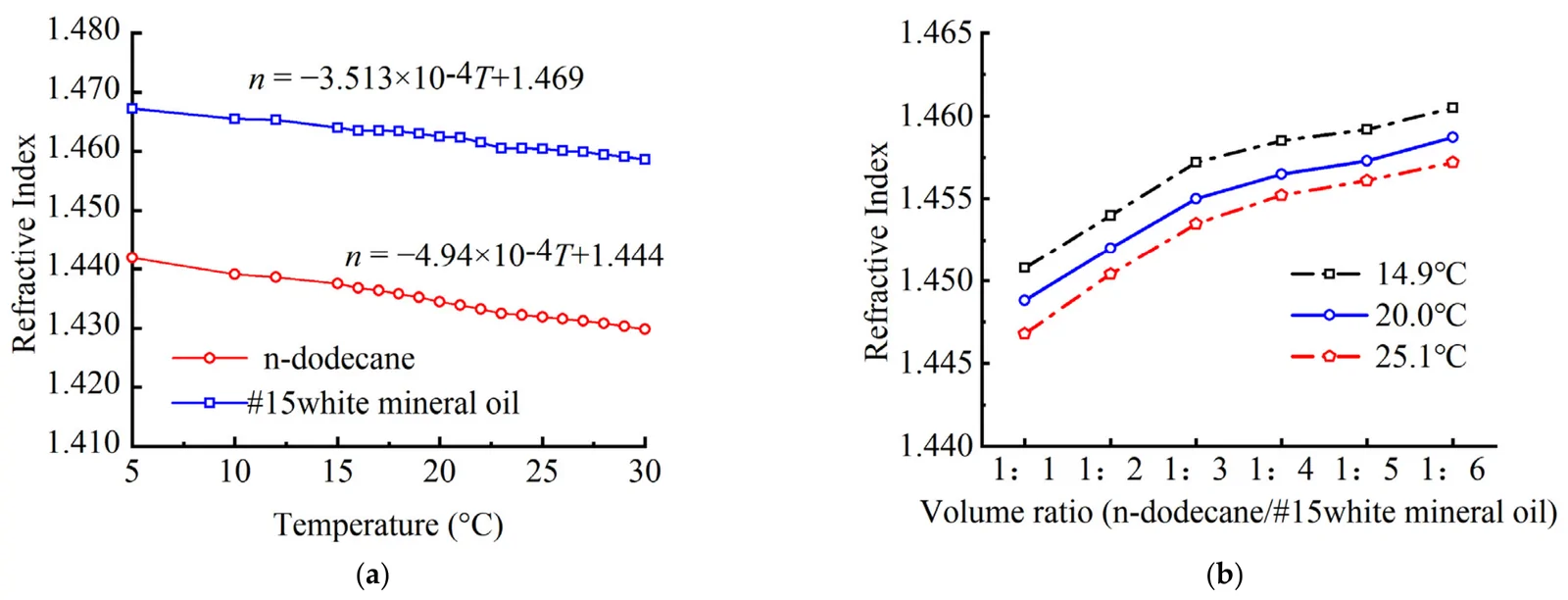
Refractive index variation of the liquid mixture with temperature. (**a**) Temperature-refractive index relationships of different mineral oils. (**b**) Volume ratio-refractive index relationships at different temperatures.

Based on the preliminary refractive-index predictions of mixed pore fluids calculated from Equation (1), a series of calibration tests were carried out, and the measured experimental data are presented below. Experimental data (Figure 1a) demonstrate that temperature exerts a significant regulatory effect on the refractive index of oil phases. Within the temperature range of 5–30 °C, both n-dodecane and #15 white mineral oil exhibit a linearly decreasing trend in refractive index with increasing temperature. For mixed oil phase systems under constant temperature conditions, the refractive index shows a monotonically increasing trend with higher white mineral oil volume fraction, though the rate of increase displays nonlinearly decaying characteristics. Notably, under fixed composition ratios, the temperature-dependent response pattern of the mixed liquid’s refractive index remains consistent with that observed in single oil phases (Figure 1b), which shows good agreement with the findings reported by Hu et al. [29] regarding the optical properties of mineral oil blends. Based on these temperature-sensitive characteristics, it is recommended to implement precise temperature control (±0.5 °C) during experiments to eliminate systematic errors in refractive index measurements caused by thermal fluctuations.

At the same temperature, the relationship between the refractive index of the pore solution in the mixed liquid and the volume ratio can be determined using the Lorentz formula [30].

In the equation: $n_{12}$ denotes the refractive index of the mixed pore fluid (dimensionless); $n_{i}$ (i = 1, 2) represents the refractive index of any individual fluid (dimensionless); $P_{i}$ (i = 1, 2) is the component mass fraction of any individual fluid; $\rho_{i}$ (i = 1, 2) denotes the density of any individual fluid. When two mineral oil fluids are known, the approximate volume ratio can be calculated using Equation (1).

(1) $$\frac{n_{12}^{2}-1}{\left(n_{12}^{2}+2\right)\rho_{12}}=\left(\frac{n_{1}^{2}-1}{n_{1}^{2}+2}\right)\frac{P_{1}}{\rho_{1}}+\left(\frac{n_{2}^{2}-1}{n_{2}^{2}+2}\right)\frac{P_{2}}{\rho_{2}}$$

### 2.3. Preparation Method of Graded Transparent Soil

**Figure 2:**
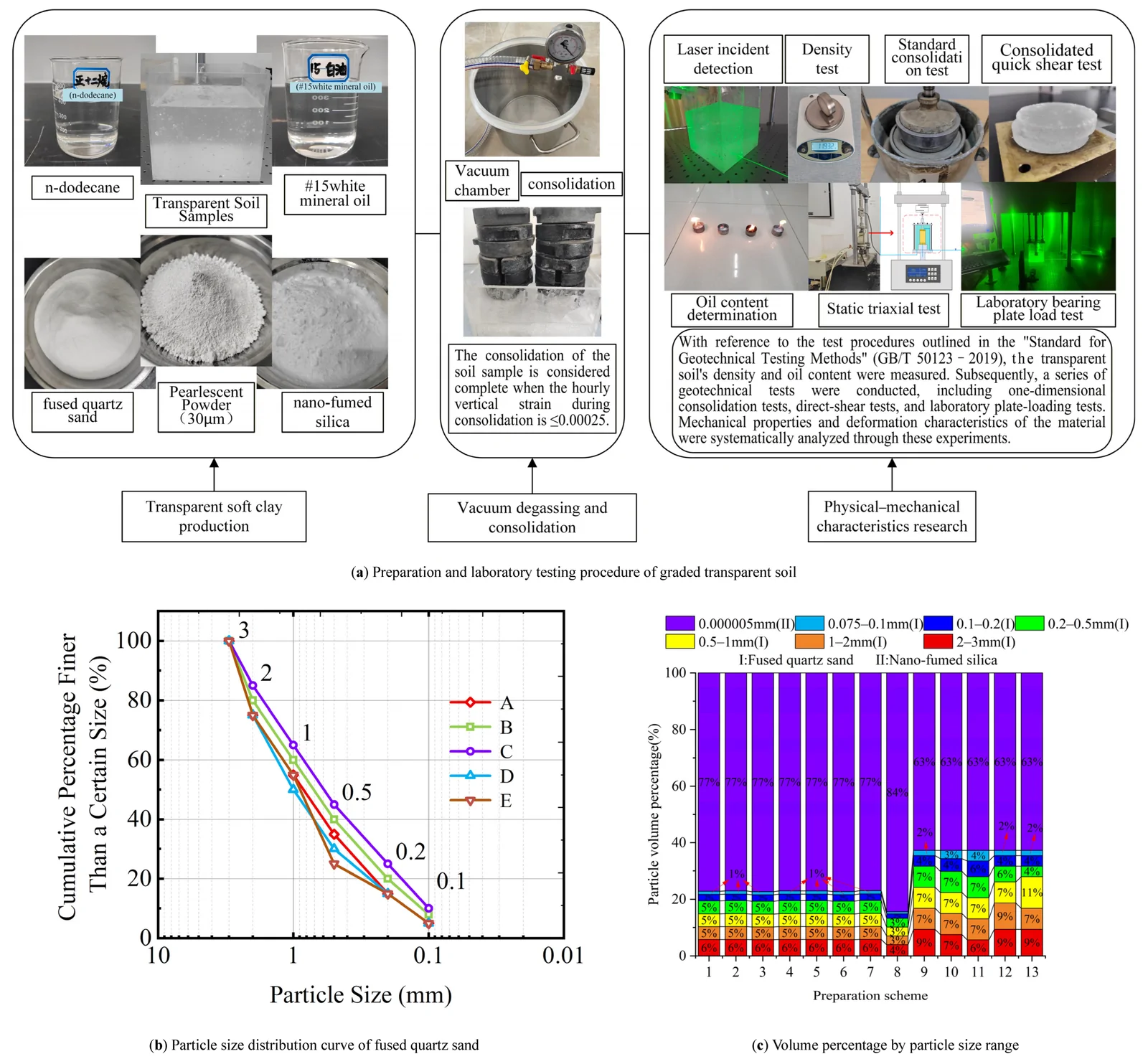
Preparation procedure of graded transparent soil and volume fraction of different particle sizes.

Material synthesis experiments were conducted based on the 13 formulation programs listed in Table 3. The experimental process required the use of a constant-temperature drying oven, Abbe refractometer, electronic balance, mechanical stirrer, custom acrylic glass test molds (with a wall thickness of 15 mm), and a vacuum degassing device. As shown in the process flow diagram in Figure 2a, the mixing and vacuum degassing steps were identified as critical procedures. The entire preparation process was carried out in a temperature-controlled laboratory environment (20 ± 0.5 °C). The specific operating procedures and technical key points are as follows:

(1) Preparation of Pore Fluid: The laboratory ambient temperature was maintained at 20 °C using an air conditioner. The blended fluid was prepared according to the formulation in Table 3. After thorough stirring, the mixture was allowed to stand for 5 min. Its refractive index was measured using an Abbe refractometer. If the measured refractive index was lower than the target value, 15# white oil was added; conversely, n-dodecane was added. The calibrated fluid was then divided into two equal volumes (1:1 ratio by volume), one for the subsequent mixing process and the other reserved as a stock solution for model filling.
(2) Material Mixing and Saturation: The fused quartz sand of various particle sizes (gradation shown in Figure 2b) was rinsed thoroughly at least three times. After cleaning, it was dried in an oven and weighed according to Table 3 before being placed into a beaker containing the prepared pore fluid. The nano-fumed silica was divided into ten equal-mass portions. These portions were gradually added to the mixture while stirring with a glass rod to form a colloidal dispersion system. Pre-treated fused quartz sand was then added to the dispersion in the designated proportion (volumetric ratios of fused quartz sand to nano fumed silica are detailed in Figure 2c). The mixture was continuously stirred using a mechanical stirrer until a transparent, viscous substance was formed.
(3) Vacuum Saturation: The prepared mixture was carefully transferred into a model container measuring 200 mm × 200 mm × 400 mm without compaction to avoid damaging the pore structure under external force. Vacuum saturation was performed in two stages: First, 50% of the reserved stock solution was introduced, and vacuum treatment was applied at a negative pressure of −0.1 MPa for 1 h. Subsequently, the remaining liquid was added, and vacuum infiltration continued for an additional 2 h.
(4) De-oiling and Consolidation: Qualitative filter paper with a pore size of 1–3 μm, saturated with the blended fluid, and an acrylic loading plate were placed gently on the surface of the slurry. Weights were then placed incrementally on the pressure plate to apply graded loading, facilitating the expulsion of excess fluid and consolidation of the mixture, thereby completing preparation of the transparent soil.

The specific formulation is shown in Table 3.

## 3. Visual Evaluation of Similar Materials

### 3.1. Evaluation Method

Each transparent soft clay specimen was placed in an acrylic glass model box with identical width (100 mm). The back panel of the model box was affixed with an A4 sheet printed with textual characters (“transparent soft clay”). Under standardized imaging conditions (consistent shooting angle and distance), a CCD industrial camera was employed to capture clarity images of the text visible through the transparent soft clay. The transparency effectiveness was evaluated based on both the observable text clarity in the images and the smallest discernible font size that could be visually identified.

### 3.2. Evaluation Results

**Figure 3:**
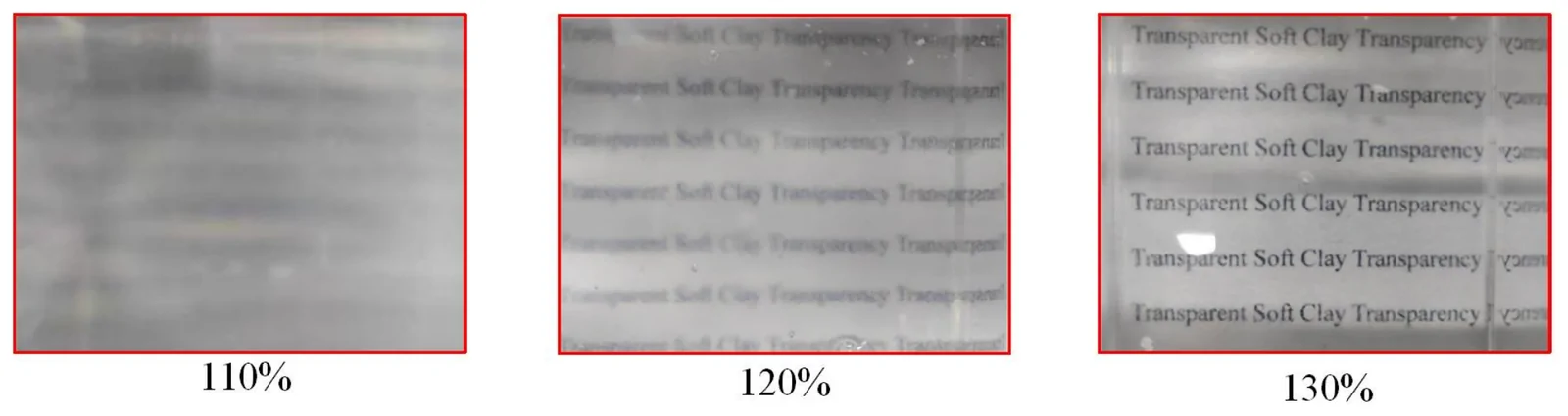
Visibility of transparent soil under varying oil-to-soil ratios.

**Figure 4:**
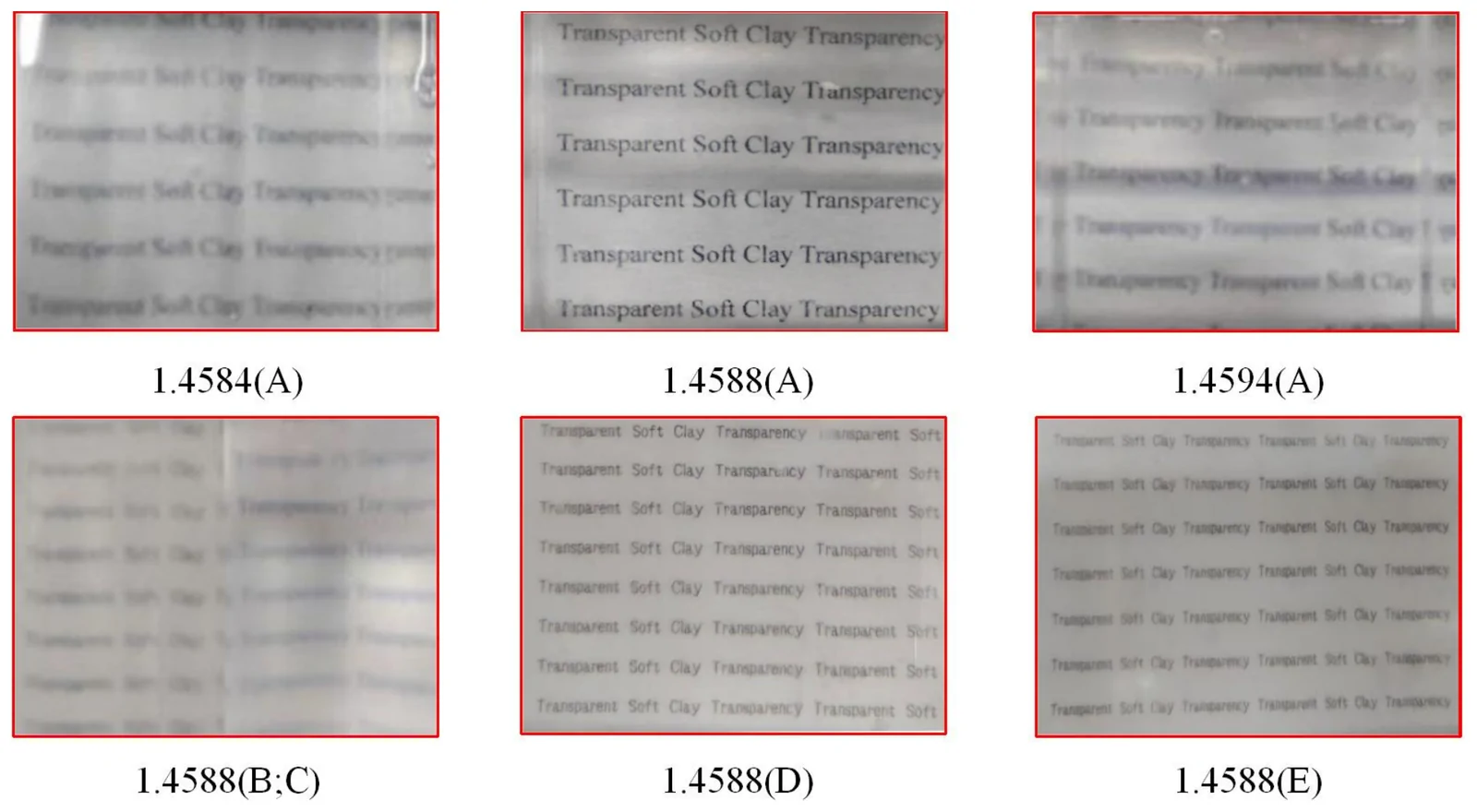
Visibility of transparent soil under different refractive indices.

In each scenario, a pre-consolidation pressure of 25 kPa was applied to the transparent soft clay until it was stable and around 100 mm thick, followed by sequential optical measurements on the specimens. Figure 3 and Figure 4 illustrate the influence of variations in the oil-to-soil mass ratio *m*_1_/(*m*_2_ + *m*_3_) and medium refractive index on the light transmittance characteristics of the material, respectively. The oil-to-soil ratio parameter represents the proportional relationship between the mass of the oil phase and the total mass of the solid soil phase.

From Figure 3, it can be observed that under the same conditions of other variables, the nano-fumed silica content significantly affects the visibility of transparent soft clay. The optimal visualization effect occurs when the oil-to-soil ratio reaches 130%. This is because the nano-fumed silica content influences the refractive index matching between solid and liquid phases in the transparent soft clay system. Meanwhile, a higher oil content facilitates the formation of continuous oil films that encapsulate solid particles, reducing interfacial reflections between particles. Under the same mass conditions, transparent soil specimens prepared with gradations (A, D, E) exhibited better visualization effects compared to those with gradations (B, C). The poorer performance of gradations (B, C) is attributed to excessive fine particle content (0.015–2 mm), which leads to decreased void ratio and insufficient pore fluid saturation. This increases light scattering and reduces transparency. Additionally, smaller particle sizes result in more residual air bubbles and lower pore fluid saturation. This is also why transparent soft clay generally exhibits lower transparency than transparent sandy clay.

**Figure 5:**
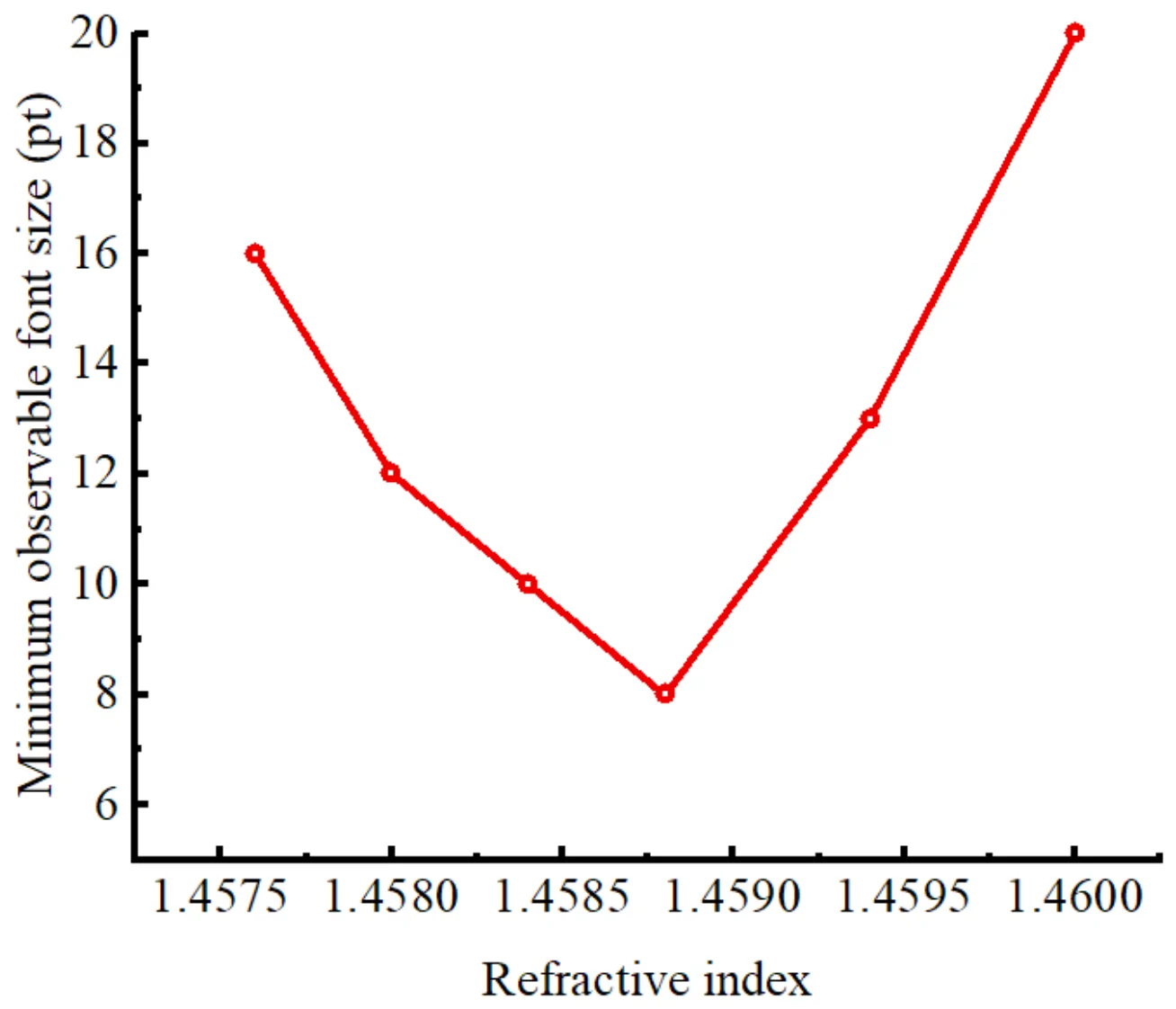
Relationship curve between refractive index and minimum font size.

This study employed the critical visual font size as an indicator to systematically evaluate the influence mechanism of refractive index on the visualization effectiveness of transparent media. As shown in Figure 5, experimental data indicated that when the refractive index reached 1.4588, the transparent soil system exhibited optimized optical visualization characteristics. This result suggests that under this ratio condition, favorable refractive-index matching was achieved between the pore liquid and solid-phase medium, thereby significantly improving the interfacial visibility of the multiphase media system. To further eliminate the subjectivity of visual font-size judgment and quantitatively verify the optical performance, a supplementary image grayscale and intensity analysis was performed on all transparent soil samples. All visual images were captured under consistent light intensity, shooting distance, and background conditions to ensure unified testing standards. The average grayscale intensity value and image uniformity were extracted and statistically compared for each refractive index condition. The results demonstrate that the sample with a refractive index of 1.4588 yields the highest average light intensity and the most uniform grayscale distribution, indicating minimal light scattering and interfacial reflection. This quantitative optical evidence further verifies that the matched refractive index effectively reduces optical heterogeneity and provides superior internal visual clarity for subsequent model test observations.

## 4. Verification of Physical and Mechanical Properties of Graded Transparent Soil

Before comparing the geomechanical indices between graded transparent soil and natural soft clay, the similarity evaluation framework adopted in this study is defined herein. The simulation feasibility was assessed from two fundamental geotechnical perspectives: deformation similarity and strength similarity. Deformation characteristics were evaluated based on the compression coefficient $\alpha_{v}$ and the constrained modulus $E_{S}$, whereas shear-strength similarity was verified by cohesion *c* and the internal friction angle φ. Good agreement of these key parameters was regarded as the primary criterion to judge whether the developed transparent-soil material can reproduce the mechanical behavior of natural soft clay under the test conditions.

### 4.1. Density and Oil Content

As shown in Table 4, four groups of transparent soft clays with different refractive indices (each group has the same gradation of fused quartz sand) were used for this study, and their densities and oil contents were determined. When determining the oil content in transparent soil, the conventional oven-drying method proved ineffective for complete evaporation due to the high boiling point of white oil. Gong Quanmei et al. [12], considering the flammability of both #15 white mineral oil and n-dodecane, employed an alcohol burning method for oil content measurement, successfully controlling the oil content error within 5.00%. The calculation formula (Equation (2)) involves three parameters: representing the mass of the aluminum container, the total mass, and the residual mass. This study adopted the same methodology for oil content verification.

(2) $$w_{1}=\frac{m_{2}-m_{3}}{m_{3}-m_{1}}\times 100\%$$

As shown in Table 4, with four sets of repeated density test data, the apparent density of specimens obtained using the ring knife method ranged from 1.21 to 1.26 g/cm^3^. This indicates that the density of transparent soil will remain within a specific range as long as the ratio of pore fluid to solid particles remains constant during preparation. Its density is equivalent to approximately 61.2% of that of natural clay. This significant discrepancy stems from the micron-scale capillary network structure developed between silica particles, which endows the material with exceptionally high oil absorption capacity [26,27]. When conducting indoor model tests, particular attention must be paid to controlling the similarity ratio between the material density parameters and those of natural soil.

### 4.2. Compressive Behavior of Graded Transparent Soil

The test objects for physical and mechanical properties were selected from the specimens configured in Scheme 9 in Table 3. Prior to conducting the compression characteristics test, a pre-consolidation stress of 25 kPa was applied to the specimens using a graded loading method until the hourly vertical strain stabilized below 0.00025%. Subsequently, the soil sample collected using a cutting ring was placed in the oedometer, and a consolidation pressure of 25 kPa was applied. This prestress continued until the specimen stabilized. The standard consolidation test was then performed in strict accordance with the “Standard Test Methods for Geotechnical Testing” GB/T 50123-2019 [31].

**Figure 6:**
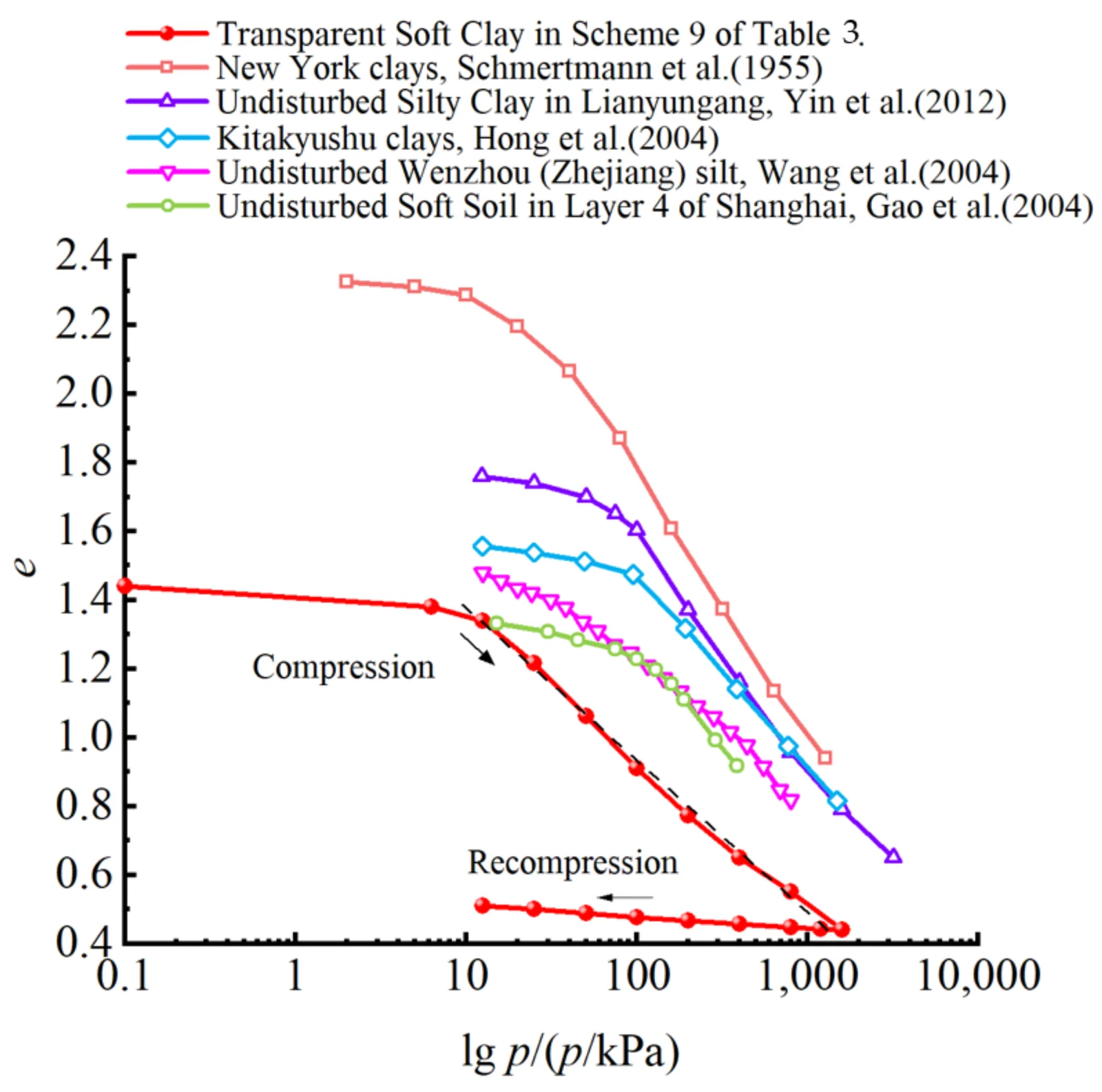
Comparison of e-lgp curves between graded transparent soil and soft clays from different regions [32,33,34,35,36].

The experimental results indicate that the transparent soft clay exhibits a compression coefficient ($\alpha_{v}$) of 1.43 and a compression modulus ($E_{S}$) of 1.72, which align with the characteristic indices of highly compressible soils. Here, $\alpha_{v}$ quantifies the void ratio change under a unit vertical stress increment within the adopted 0.1–0.2 MPa range, whereas $E_{S}$ reflects the overall vertical compressive stiffness over the same pressure interval. For this graded transparent soil composite, consisting of a fused quartz sand skeleton bonded by nano fumed silica in a mixed alkane pore fluid, these two indices collectively represent the integrated volumetric compressibility of the solid–liquid multiphase structure and are consistent with the typical parameters of highly compressible natural soft clay. By integrating the current test data with existing research findings, a comparative framework of void ratio versus logarithm of pressure (*e*-*lgp*) curves between transparent soft clay and natural soft clay was established (Figure 6). A systematic comparison of their key mechanical parameters is presented in Table 5.

The curve analysis indicates that the soil exhibits a significant linear decay trend when the pressure exceeds a certain stress threshold. Due to differences in soil composition and structure, as well as the influence of pre-consolidation pressure, the structural characteristics of the soil demonstrate a “flat initial segment followed by a steep latter segment” pattern. This curvilinear variation is similar to the segmented variation observed in marine soft clays, such as the undisturbed silty soft clay in Shanghai’s Layer ④, which shows exceptional sensitivity to stress variation processes.

### 4.3. Shear Strength Characteristics of Graded Transparent Soil

#### 4.3.1. Shear Strength Parameters

**Figure 7:**
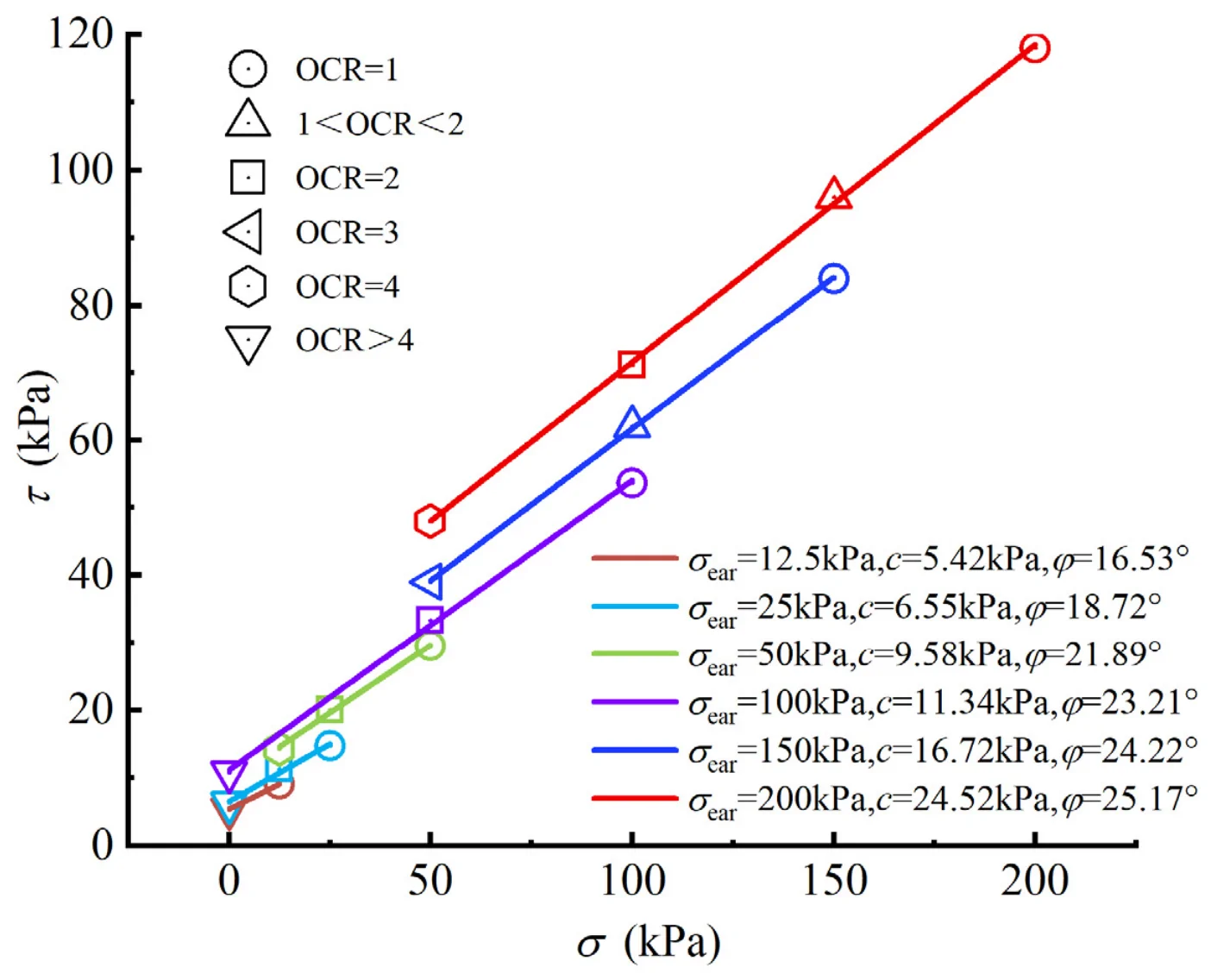
Shear strength envelopes of soft clay under different pre-consolidation pressures.

Using a strain-controlled direct shear apparatus (ZJ-D), consolidated-quick shear tests under different stress states were conducted on transparent soft clay with pre-consolidation pressures ranging from 12.5 to 200 kPa at a shearing rate of 0.8 mm/min, in accordance with GB/T 50123-2019, Standard for Geotechnical Testing Methods. During sample preparation, a consolidation pressure ($\sigma_{ear}$) of 12.5 kPa was first applied until stabilization. Subsequently, samples were obtained using a ring knife and were transferred to the direct shear apparatus for incremental loading to the target pre-consolidation pressure. Through the analysis of experimental data, a strength envelope diagram was constructed, with normal compressive stress (σ) as the abscissa and peak shear stress (τ) as the ordinate (Figure 7).

Quantitative analysis reveals that both cohesion (*c*) and the internal friction angle (*φ*) exhibit significant positive correlations with pre-consolidation pressure, with cohesion demonstrating higher sensitivity to pre-consolidation pressure variations. This study found that under lower pre-consolidation pressures (<100 kPa), cohesion (*c*) ranges between 5.42–11.34 kPa, whereas the internal friction angle (*φ*) varies within 17–23°. When pre-consolidation pressure reached or exceeded 100 kPa, cohesion showed marked enhancement with increasing pressure, ranging from 23° to 25°. A comparative analysis with undisturbed soft clay data from Tianjin Binhai area [37] confirmed good equivalency in the strength parameters between the test material ($\sigma_{ear}$ = 50 kPa) and natural soil, validating its effectiveness in simulating typical soft clay’s physical–mechanical characteristics.

#### 4.3.2. Characteristics of Shear Stress vs. Shear Displacement

**Figure 8:**
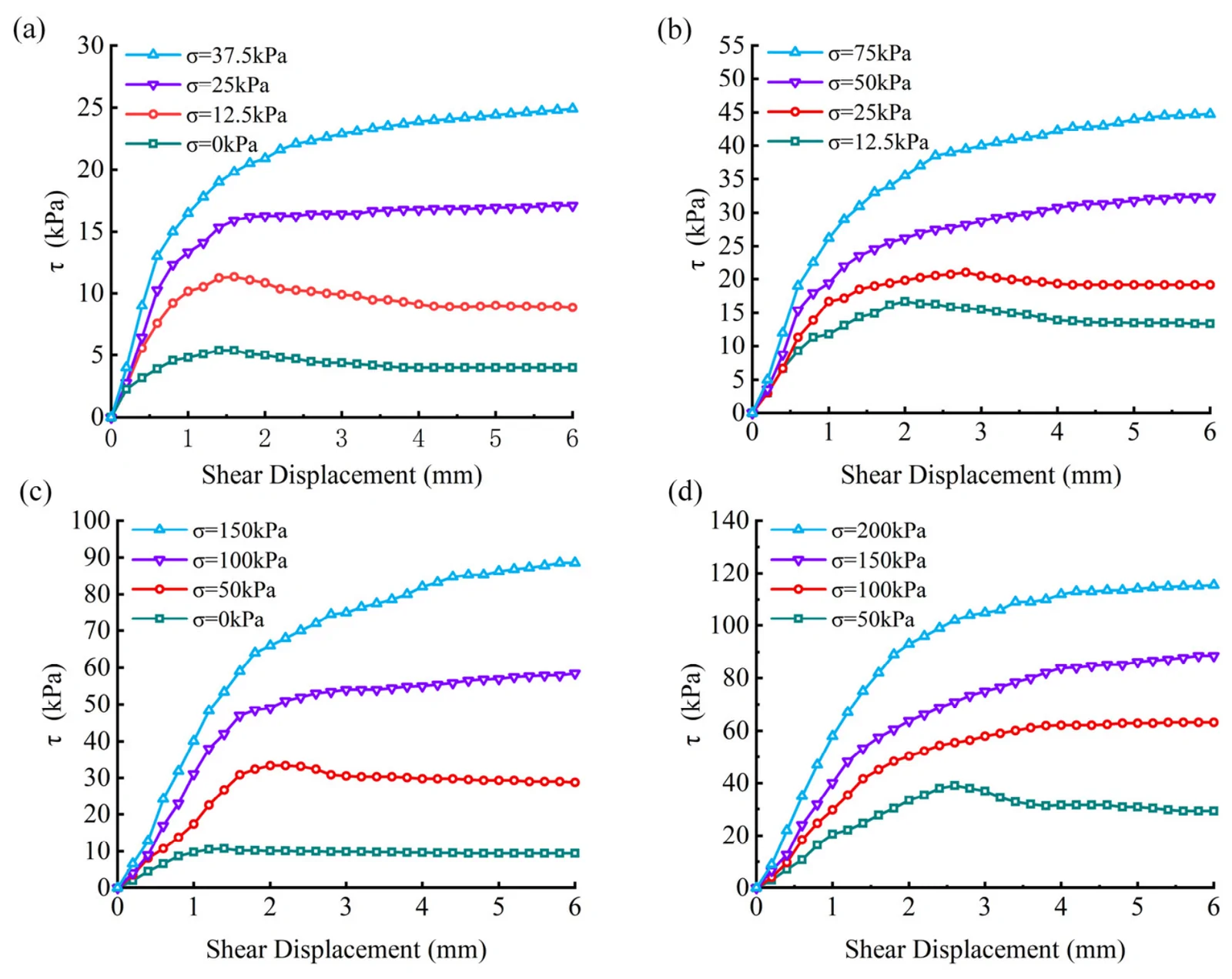
Shear stress–shear displacement relationship curves of transparent soil under different pre-consolidation pressures: (**a**) 25 kPa, (**b**) 50 kPa, (**c**) 100 kPa, and (**d**) 150 kPa.

To better characterize the shear behavior of transparent soft clay, this study presents the shear stress versus shear displacement curves of transparent soft clay specimens under different pre-consolidation pressures (Figure 8). Experimental results demonstrate that transparent soft clay shares identical evolutionary patterns with natural sedimentary clays during shearing. Under normal consolidation conditions, the material exhibits typical strain-hardening characteristics. In over-consolidated states, specimens undergo four distinct phases: elastic deformation, plastic deformation, softening, and a residual stage, ultimately stabilizing at a residual strength determined by interparticle friction. However, under specific conditions ($\sigma_{ear}$ = 150 kPa with vertical stress = 100 kPa), the curve displays slight hardening rather than softening. This anomaly is attributed to the relatively low over-consolidation ratio, structural changes during post-consolidation sampling, and stress release during unloading to the target vertical stress level for testing.

### 4.4. Triaxial Test

Although the shear-strength performance of transparent soil has been preliminarily verified by direct-shear test results compared with published muddy-clay data, triaxial tests were further performed in this section. Unlike direct-shear comparisons against literature data, triaxial results enabled a direct benchmark with undisturbed muddy-clay samples collected from Shaoxing, offering a more comprehensive and in-situ-oriented evaluation of the shear behavior of our transparent soil simulant.

According to the testing procedures specified in the Standard for Geotechnical Testing Methods (GB/T 50123-2019), graded transparent soil specimens with a pre-consolidation pressure of 12.5 kPa were prepared by cutting. Systematic comparisons of void ratio and compressive characteristics between the developed transparent soil and multiple natural soft clays were completed in the one-dimensional compression section and were not repeated here. Two groups of specimens were prepared, with four specimens in each group corresponding to confining pressures of 100 kPa, 200 kPa, 300 kPa, and 400 kPa, respectively. Consolidated–drained (CD) triaxial compression tests were conducted on each specimen at a low shearing rate of 0.0022 mm/s.

Notably, no water was involved in the specimen saturation process. The transparent soil was fully saturated with mixed n-dodecane and 15# white oil throughout sample preparation and testing. The deionized water in the triaxial cell only acts as the confining pressure medium and is completely isolated from the oil-saturated specimen by a latex membrane, without any infiltration or fluid mixing. Due to the oil-based non-aqueous pore fluid, the traditional Skempton B-value test designed for water-saturated soils is theoretically inapplicable in this study. Full saturation was reliably achieved through vacuum degassing, long-term oil immersion, and stepwise low-pressure pre-consolidation to eliminate residual air bubbles.

Unlike natural water-saturated soft clay, the developed transparent soil possesses oil pore fluid with higher viscosity and lower permeability. Therefore, this ultra-low shearing rate was specifically adopted to provide sufficient time for the internal oil-based pore fluid to drain gradually and dissipate residual pore pressure during shearing, ensuring strict fully drained conditions and accurate calibration of effective shear strength parameters. The external cell water only provides uniform confining pressure and does not participate in pore drainage or affect the internal mechanical response of the material. The test results were compared with those of intact muddy clay sampled in Shaoxing.

**Figure 9:**
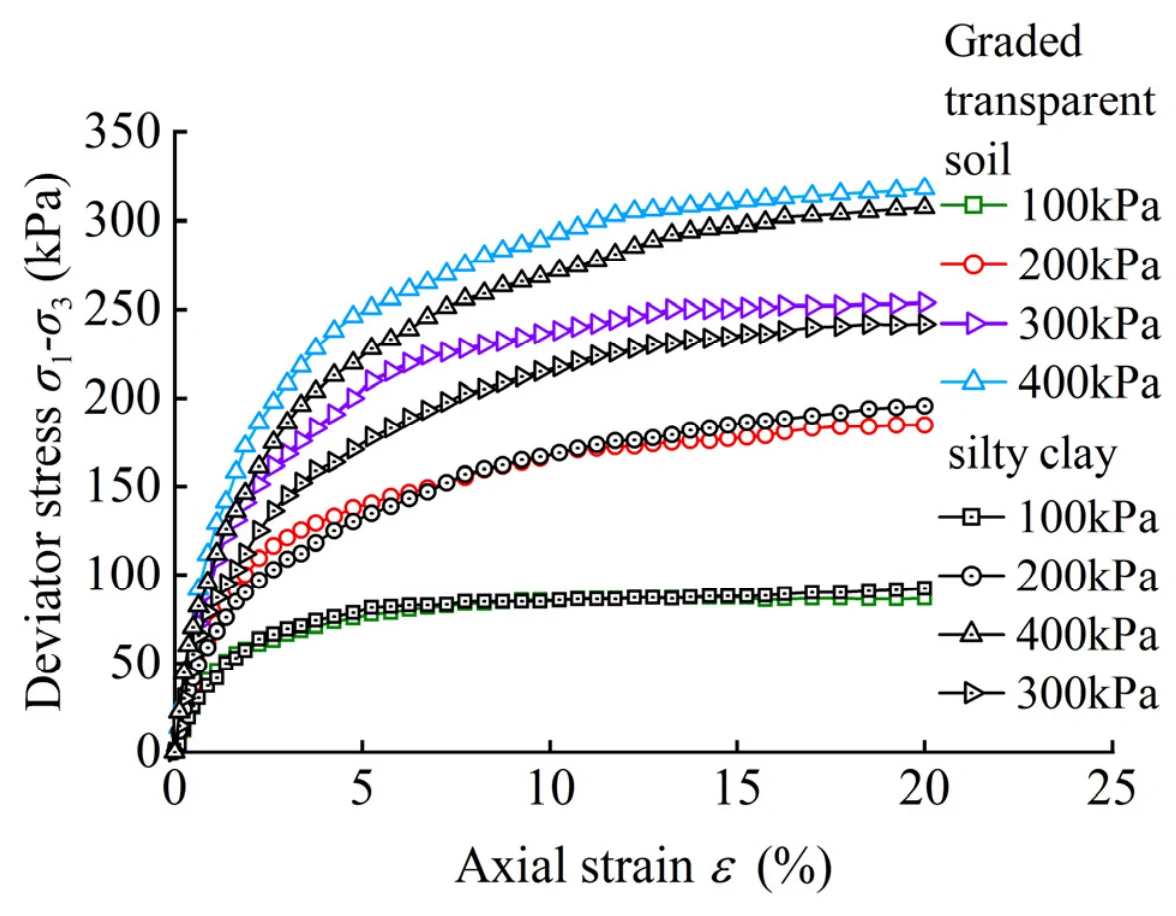
Deviatoric stress–axial strain curve.

All CD triaxial tests in this study adopted a standard stress path of isotropic consolidation followed by constant-confining-pressure drained shear. Specimens were first isotropically consolidated under target confining pressures and then sheared steadily with constant confining pressure throughout the loading process. The triaxial compression test results indicate that the stress–strain curves of the graded transparent soil and the Shaoxing silty clay under the four confining pressures (100 kPa, 200 kPa, 300 kPa, and 400 kPa) show similar strain-hardening deformation patterns compared to soft clay. Both soils clearly demonstrate an elastic stage followed by a plastic deformation stage. In the small-strain stage, the deviator stress increases linearly with strain at a rapid rate. In the large-strain stage, the soil enters the plastic state, where the deviator stress increases nonlinearly with a gradually slowing rate until it stabilizes. No distinct peak stress or yield failure point is observed, showing typical strain-hardening steady-state deformation without strain softening. Meanwhile, both materials exhibit comparable strengthening responses to increasing confining pressure, as shown in Figure 9. Under all four confining pressure levels, the relative error in the ultimate deviator stress between the two soils is less than 5%.

**Figure 10:**
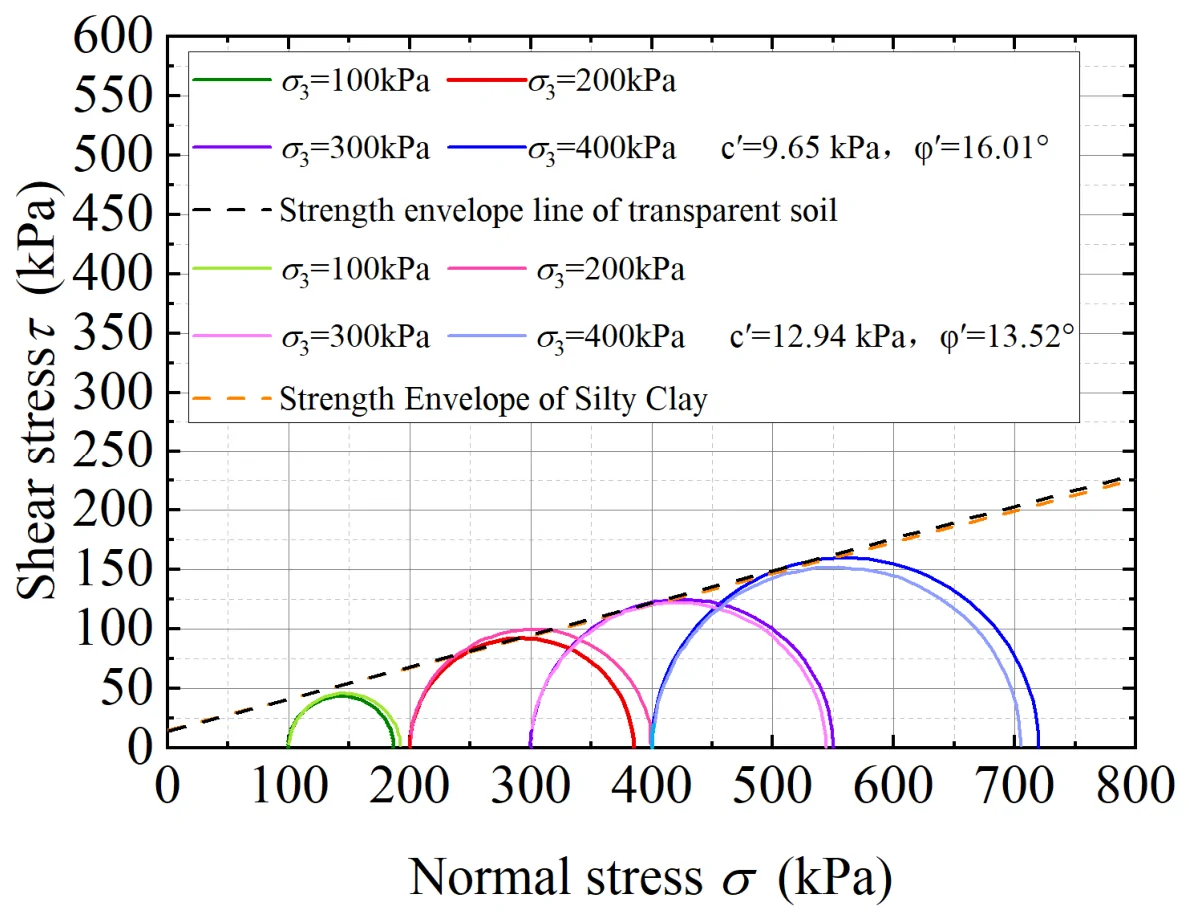
Shear strength envelope from the triaxial test.

Based on the stress values under different confining pressures, Mohr’s stress circles were plotted, and strength envelopes were fitted, as shown in Figure 10. All strength parameters derived from CD triaxial tests were effective stress indices. The effective shear strength parameters obtained for the graded transparent soil were cohesion c′ = 9.65 kPa and the internal friction angle φ′ = 16.01°, whereas those for the Shaoxing silty clay were c′ = 12.94 kPa and φ′ = 13.52°. The Mohr–Coulomb strength envelopes of the two soils were similar, with both sets of shear strength parameters falling within the typical range for soft clay. The internal friction angles are highly comparable, whereas the difference in cohesion stems from the inherent material properties and can be reasonably explained. Therefore, the graded transparent soil exhibits comparable mechanical behavior to the natural Shaoxing silty clay and can serve as a promising simulative material for modelling natural soft clay.

Mechanically, despite the angular morphology of graded fused quartz particles that may cause dilatancy in pure granular materials, the fumed silica colloidal coating effectively isolates particle interlocking. During shear loading, the transparent soil predominantly exhibits volumetric contraction rather than dilatancy, which conforms to the fundamental contractive feature of normally consolidated soft clay. Furthermore, the synthetic oil-silica matrix exhibits rate-dependent viscoplastic characteristics similar to natural soft clay. The nano-fumed silica forms a stable colloidal network in the oil phase, endowing the material with structural viscosity and stress relaxation properties, whereas the viscous oil pore fluid produces time-dependent creep and slow drainage deformation. Such rheological behaviors are consistent with the typical creep and stress–relaxation responses of natural soft clay under quasi-static loading, further verifying the rationality of the adopted low shearing rate and the feasibility of the transparent soil for soft ground simulation.

## 5. Application of Graded Transparent Soil in Model Tests

### 5.1. Plate Load Model Test

**Figure 11:**
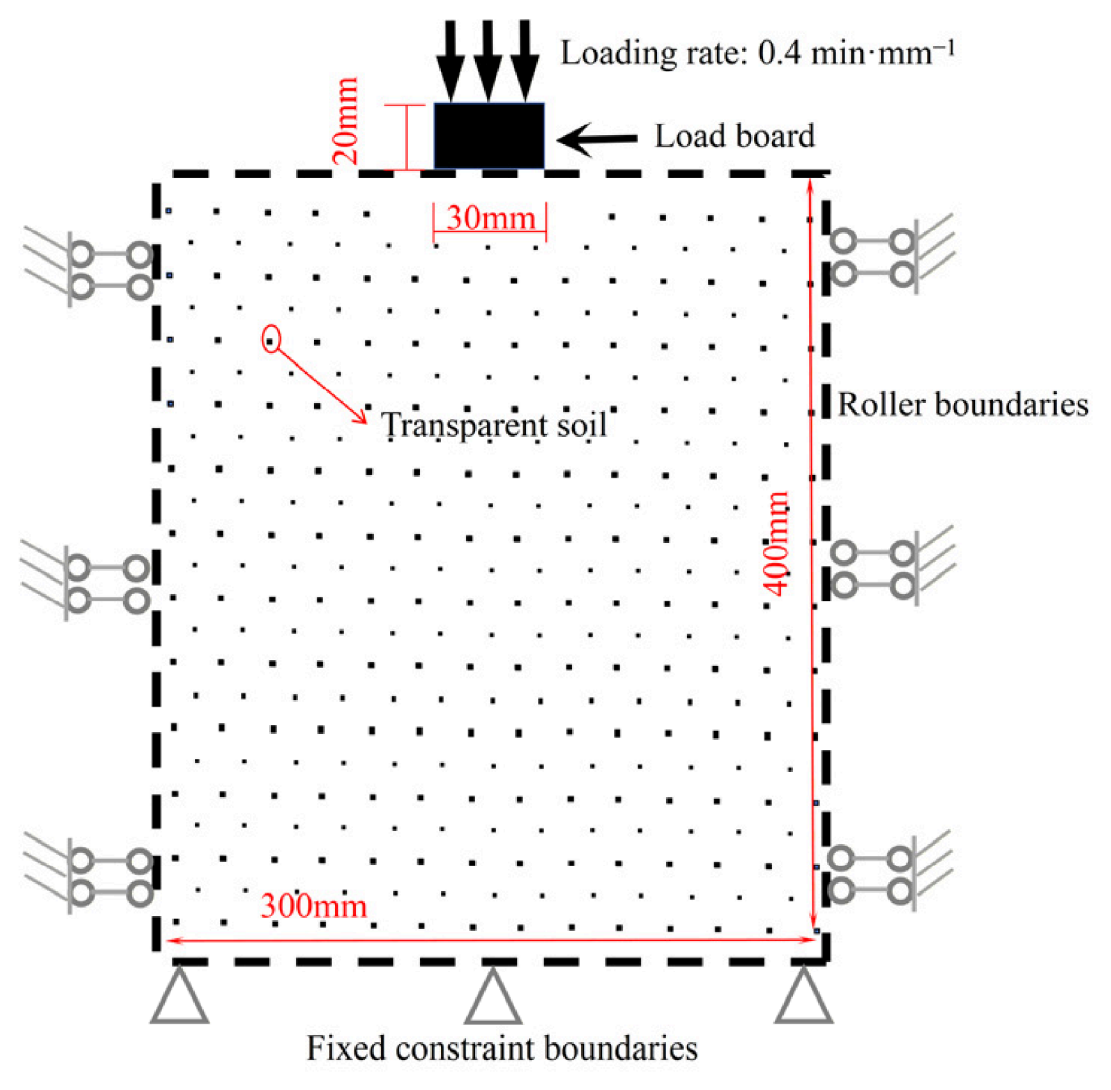
Schematic diagram of the model test.

**Figure 12:**
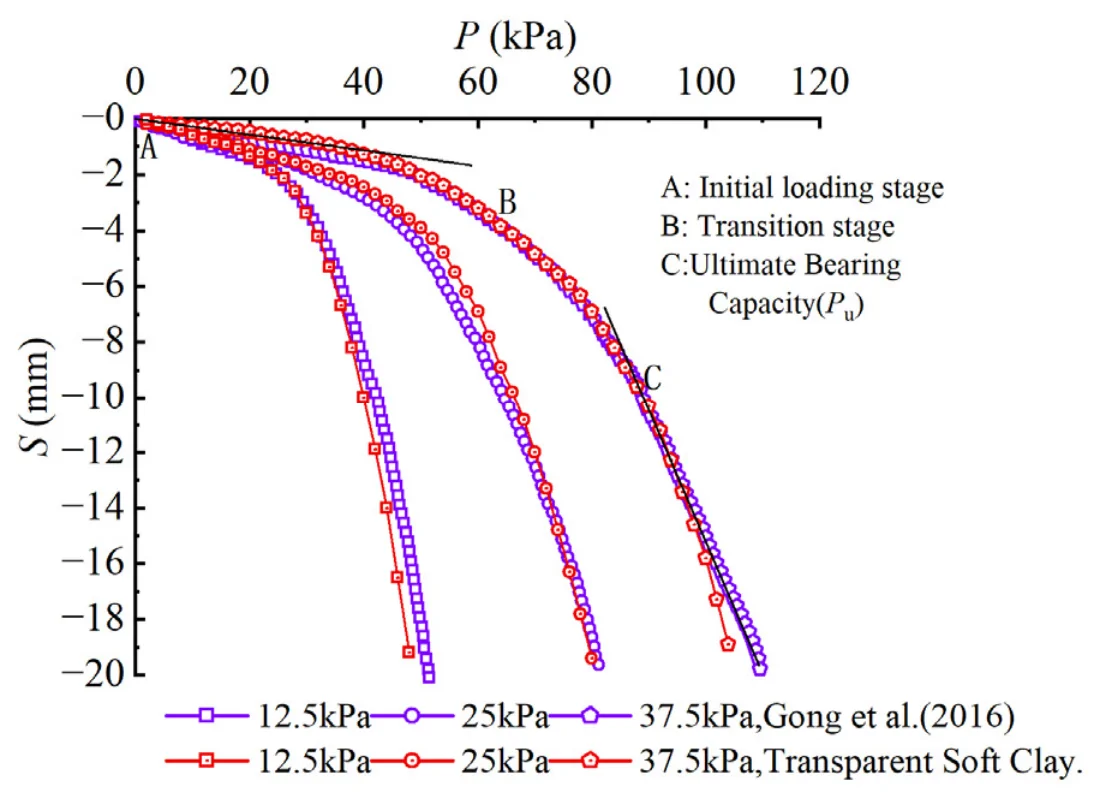
Load–settlement curve under three pre-consolidation pressures for transparent soft clay, compared with the test results reported by Gong et al. [12].

The plate load model test can accurately reflect the bearing capacity characteristics and settlement development patterns of transportation subgrades, abutment foundations, and soft soil foundations at stations under vehicle loads, structural self-weight, and additional stresses. It serves as a core method in geotechnical engineering for evaluating foundation deformation mechanisms. In this study, indoor plate load tests with a constant settlement-rate loading scheme were performed on graded transparent soil prepared in accordance with Formula 9. The test setup is illustrated in Figure 11, and the overall experimental procedure is presented in Figure 2. The load-settlement (*P*-*S*) curves obtained from the plate-loading test are shown in Figure 12. This experimental scheme was adopted to validate the feasibility of the graded transparent soil for simulating natural soft clay, by analyzing the macroscopic bearing performance and microscopic failure mode of foundation soil.

Different from conventional plate load tests that only record macroscopic load-settlement (*P*-*S*) responses, this study further incorporates the Particle Image Velocimetry (PIV) technique to realize the full-field internal deformation monitoring of foundation soil. During the continuous loading process, high-resolution sequential images of the transparent soil cross-section were synchronously acquired, and post-processed to derive the internal displacement vectors and strain field distribution of the soil mass. The core purpose of introducing PIV monitoring is to quantitatively characterize the subsurface deformation evolution, visualize the development of progressive sliding zones and failure boundaries, and reveal the mesoscopic foundation failure mechanism, which cannot be captured by traditional macroscopic *P*-*S* test data alone.

Multiple types of test data, including the macroscopic *P*-*S* curve, as well as the mesoscopic soil velocity field and displacement field obtained from PIV monitoring, were comprehensively compared and analyzed with results from existing studies. Here, P represents the average compressive stress at the base of the loading plate and S denotes the settlement of the loading plate. The integrated analysis of macroscopic bearing responses and mesoscopic deformation characteristics further verifies that the developed transparent soil can reliably simulate the mechanical deformation behavior and failure characteristics of natural soft clay foundations.

**Figure 13:**
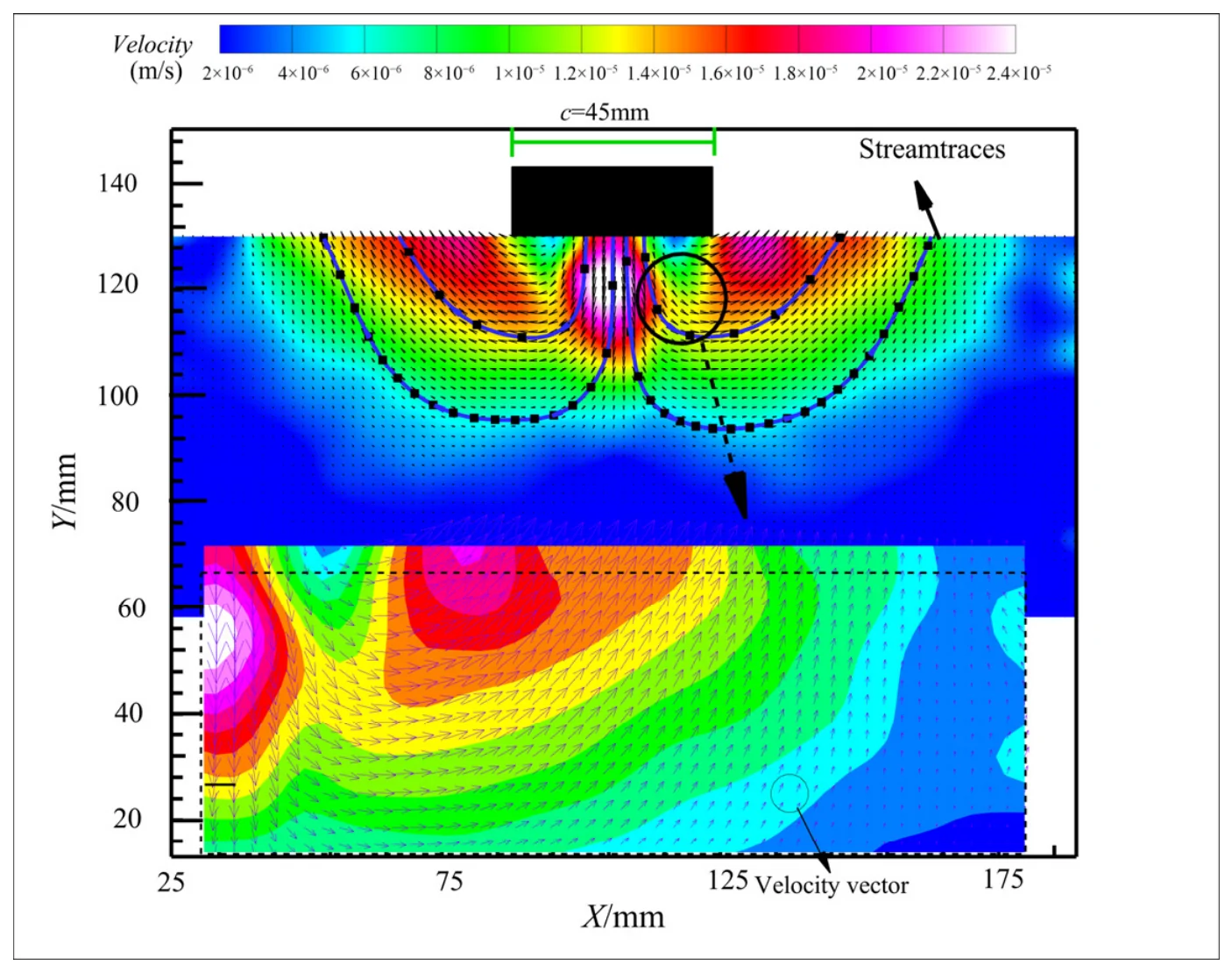
Velocity field of the model test.

**Figure 14:**
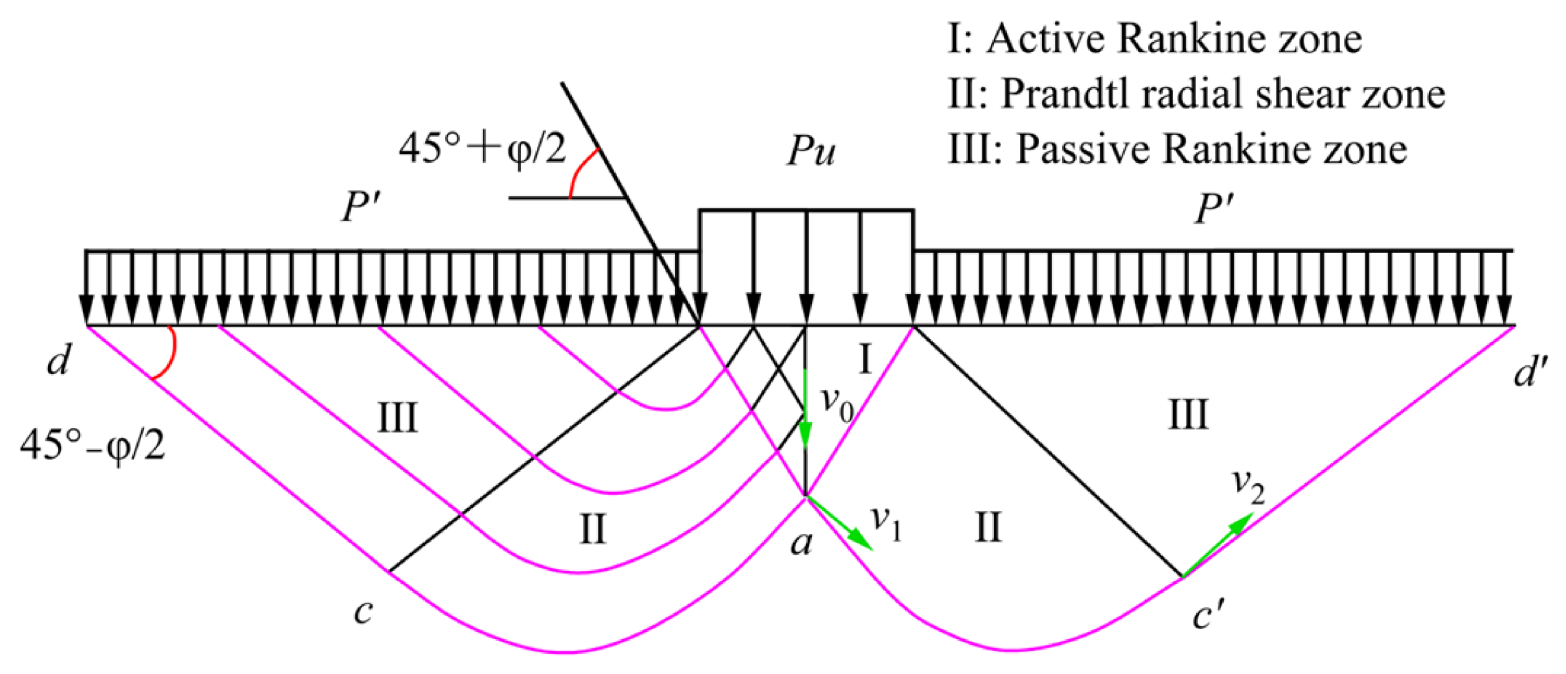
Prandtl failure pattern.

The analysis reveals that under the three different pre-consolidation pressures, the velocity fields of the soil during the settlement of the loading plate exhibit similar distribution characteristics. Based on this commonality, graded transparent soil under a representative pre-consolidation pressure of 25 kPa was selected for detailed analysis. Through post-processing analysis using Particle Image Velocimetry (PIV) technology, the distribution characteristics of the velocity field within the model test soil when the loading plate settlement reached 12 mm are shown in Figure 13. Figure 14 illustrates the foundation failure pattern according to Prandtl’s theory, where $P^{\prime }$ represents the uniformly distributed surface load, *v*_0_ is the movement velocity of the rigid block in Zone I, *v*_1_ is the slip velocity at the interface between Zone II (the non-uniform deformation zone) and Zone I relative to the surrounding soil, and *v*_2_ corresponds to the displacement velocity of the rigid block in Zone III. By comparison, it can be seen that the distribution pattern of the velocity field obtained from the plate load model test on graded transparent soil closely aligns with the theoretical velocity field of the Prandtl failure pattern. However, slight differences are observed in the measured soil velocity behavior within Zones II and III, corresponding to Prandtl’s theory: the velocity decreases significantly with increasing distance from the loading plate. This attenuation phenomenon is primarily attributed to the actual local shear failure mode that occurred during the test. Complete slip surfaces did not form in Zones II and III, indicating that the soil was in a state of non-limit equilibrium. The results of the constant-settlement-rate plate load test conducted in this study show that the load–settlement curve of the graded transparent soft clay is highly consistent with the characteristics of natural soft soil foundations. The internal displacement and velocity field distributions of the soil exhibit a typical Prandtl failure pattern. This is fully aligned with the mechanisms of plastic zone expansion and local shear failure observed in soft soil subgrades under actual traffic loads.

### 5.2. Jacked-Pile Penetration Model Test

Jacked piles are a widely adopted foundation form for soft soil grounds. The soil-squeezing effect, pile–soil interaction, and the evolution of resistance during the pile-sinking process directly determine the bearing performance and long-term stability of pile foundations. In this jacked-pile penetration model test, three working conditions were established, with the specific test scheme detailed in Table 6. All test specimens were subjected to a constant loading rate of 0.067 mm/s, with a jacked-pile penetration depth of 300 mm and an image acquisition frequency of 3 Hz. This loading rate was scaled from the practical in-situ pile driving rate based on the Froude similarity criterion, corresponding to a prototype construction rate of 0.2 m/min with a geometric scale of 1:50. The adopted low rate ensures quasi-static penetration without dynamic inertial disturbance, and matches the rate-dependent viscoplastic and creep characteristics of the oil-silica transparent soil, which guarantees the validity of the model test results. On the side of each test pile, six groups of BF350-3EB full-bridge strain gauges were symmetrically arranged along the pile length—six on each side, totaling twelve gauges bilaterally. To prevent interference with the displacement field, the strain gauges were attached at positions parallel to the laser profile. The strain gauges were connected via circuits to a full-bridge transmitter and were then transmitted to a computer.

**Figure 15:**
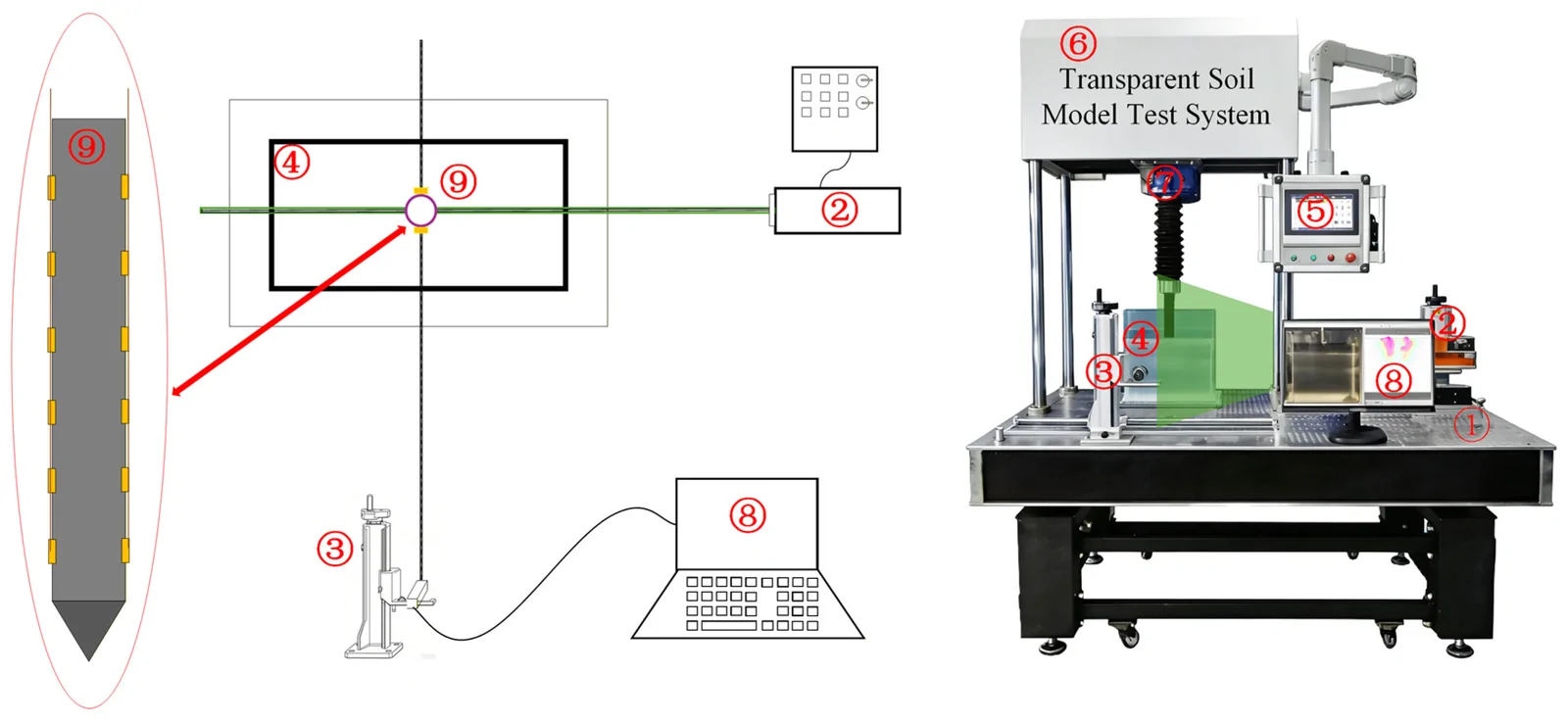
Model-test diagram of jacked-pile penetration. ① Workbench; ② Laser emitter; ③ CCD camera; ④ Model box; ⑤ Displacement controller; ⑥ Power supply box; ⑦ Sensor; ⑧ Data processor; ⑨ Model pile.

The layout diagram of the strain gauges and the jacked-pile penetration model test is shown in Figure 15. 

**Figure 16:**
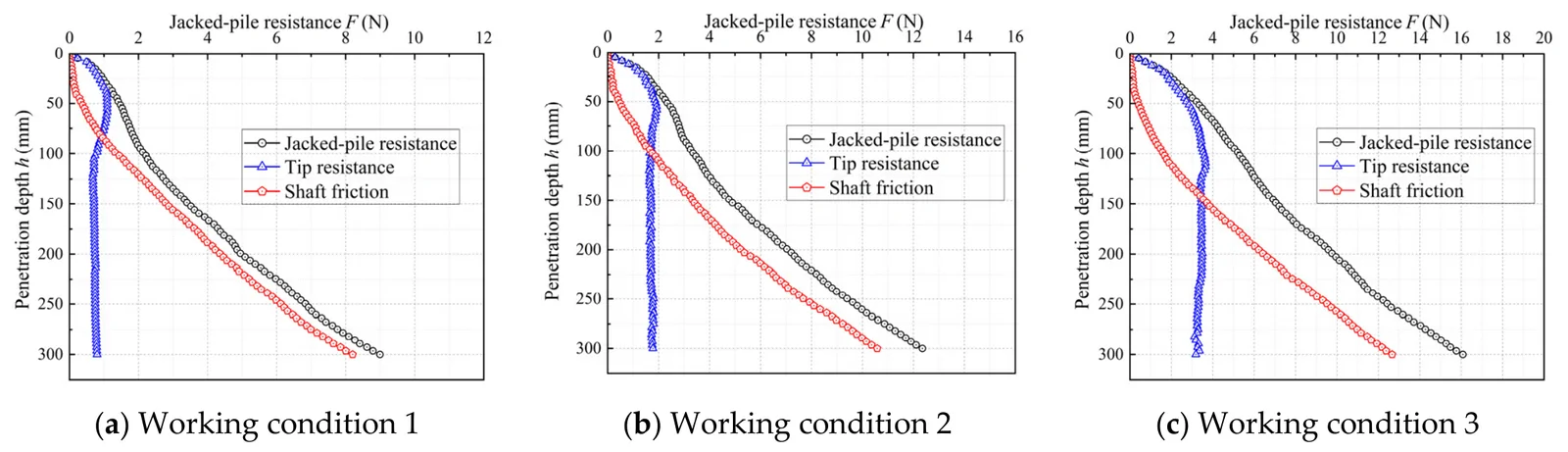
Variation curves of jacked-pile resistance with penetration depth.

Based on the jacked-pile penetration model tests, the relationship curves between jacked-pile resistance and penetration depth under three different conditions were obtained, as shown in Figure 16. The figure illustrates the evolution of shaft friction and tip resistance. From the trends of each curve, it can be observed that both the total jacked-pile resistance and the shaft friction increase progressively with greater penetration depth, whereas the tip resistance rises rapidly when the pile first penetrates the soil and then gradually stabilizes. Meanwhile, the pile diameter significantly influences the jacked-pile resistance. As the pile diameter increases, the pile–soil contact area expands accordingly, leading to higher values of total jacked-pile resistance, tip resistance, and shaft friction. Specifically, the total jacked-pile resistance increased from 8.99 N in Condition 1 to 16.08 N in Condition 3. According to the data in Table 7, in terms of resistance composition, as the pile diameter grows, the proportion of tip resistance within the total jacked-pile resistance gradually rises from 9% to 20%, whereas the proportion of shaft friction correspondingly decreases from 91% to 79%. To evaluate the pile–soil interfacial shear characteristics, interface direct-shear tests between the model pile and graded transparent soil were carried out. Under normal-stress levels of 12.5 kPa, 25 kPa, 50 kPa and 100 kPa, the measured interface friction angle was 10.88°, and the interfacial adhesion was 4.74 kPa. This interface-strength parameter falls within the typical range of interface shear properties between natural soft clay and pile surfaces. Therefore, the high proportion of shaft friction (79–91%) obtained in the static press-in pile test is the inherent mechanical response of soft-clay-stratum pile penetration, rather than being caused by abnormal frictional behavior at the pile–transparent-soil contact. Nevertheless, shaft friction consistently constitutes the main component of jacked-pile resistance, a pattern that aligns with the structural characteristic that the lateral surface area of small-diameter piles is far greater than the end-bearing area. The development trends of jacked-pile resistance with depth under different conditions are generally consistent: both total jacked-pile resistance and shaft friction exhibit a continuous linear increase with penetration depth, and shaft resistance contributes more significantly to saturated soft clay. This observation matches the results of jacked-pile penetration tests in soft soil reported by Wang Yue et al. [38].

A comparison of jacked-pile resistance from model tests under different conditions is shown in the figure below.

To further validate the rationality and reliability of the model-test observations, the experimentally obtained failure modes and deformation characteristics were compared with classic theoretical solutions and existing literature results. The observed Prandtl-type slip-failure mechanism from the plate-load test is consistent with the classic bearing-capacity theoretical framework, indicating that the transparent-soil model can reproduce the fundamental mechanical behavior of natural soft clay under foundation loading. In addition, the jacked-pile-penetration-induced soil displacement and progressive-failure patterns obtained in this study are in reasonable agreement with the qualitative and quantitative conclusions reported in previous physical model-test studies on pile penetration in soft clay [39,40,41,42]. Compared with conventional transparent-soil materials that often exhibit overly stiff or simplified failure responses [43], the developed graded transparent soil exhibits more realistic strain-development and failure-evolution features, which further demonstrates its superiority in simulating the large-deformation and progressive-failure behavior of natural soft-clay foundations. The above cross-verification suggests that the proposed material can provide feasible and physically consistent visual-test results for soft-foundation and pile-soil-interaction model tests.

## 6. Conclusions

This study presents a novel preparation method for graded transparent soil. Through comprehensive investigation of its physical and mechanical properties and model test analysis, the mechanical performance of the developed transparent soil was systematically compared with that of natural soft clay. The results indicate that the proposed material alleviates part of the mechanical limitations of conventional transparent soil for soft clay simulation. This work complements the existing material system for visual model tests of soft foundations in geotechnical engineering. The primary research findings are summarized as follows:

(1) By optimizing the gradation of fused quartz sand and utilizing the synergistic modification effect of nano-fumed silica, the internal particle skeleton of the developed material better resembles the microstructure of natural soft clay. This improved formulation alleviates the common problem of mechanical mismatch observed in traditional transparent soil simulants. The prepared material achieves favorable transparency and mechanical performance, which can serve as an alternative visual testing material for soft soil model investigations.
(2) The experimental results suggest that the compressive behavior and shear strength of the graded transparent soil are in reasonable agreement with those of natural soft clay. The mechanical strength of the material can be effectively regulated by varying the pre-consolidation pressure. The tested transparent soil is capable of reproducing the typical Prandtl failure characteristics of soft soil foundations and the force-deformation responses during jacked-pile penetration. The findings provide feasible experimental references for investigating soft foundation failure mechanisms and pile–soil interaction in geotechnical physical model tests.
(3) The proposed transparent material allows visual observation of internal deformation and progressive failure evolution in soft foundations, which improves the observation accessibility and analytical depth of physical model tests. This study provides a feasible material and technical approach for laboratory investigations of soft foundation behaviors, showing certain theoretical significance and application potential for geotechnical visualization research.

It should be noted that this study focuses exclusively on the similarity in macroscopic mechanical properties between the transparent simulant and natural soft clay and does not pursue consistency in particle size gradation or mineral composition. Meanwhile, the mechanical tests involved only cover short-term conventional mechanical behaviors, including consolidation, direct shear and triaxial tests; long-term rheological effects such as creep and stress relaxation are not within the scope of this study.

Overall, this study provides a practical preparation and optimization framework for high-transparency soft clay simulants and offers a feasible alternative material for laboratory visual investigations of soft foundation deformation and pile–soil interaction mechanisms.

## Figures and Tables

**Table 1:** Summary of typical transparent soil materials from the existing literature.

Publication Year	Authors	Type of Transparent Soil Material	Established Measured Properties	Application
2003 [7]	Liu & Iskander	Amorphous silica-oil sand	Permeability, consolidation	For sand simulation
2015 [9]	Wallace & Rutherford	Laponite water-based clay	Compression, undrained shear strength	Soft clay-related model test
2016 [10]	Gong et al.	Silica powder cohesive soil	Compressibility, shear strength	Cohesive soil visualization test
2018 [11]	Kong et al.	U10-NaOH aqueous clay	Silt compression and strength	Temperature-controlled geotechnical model test
2021 [12]	Zhou et al.	Spherical silica soil	Pile penetration mechanical response	Pile-soil interaction model test
2022 [14]	Zhong et al.	Quartz–soil–rock mixture	Shear characteristic, optical matching	Developed for soil-rock mixture test
This study	Wang et al.	Graded fused quartz sand and nano-fumed silica	Compression index, shear strength, high transparency	Adjustable strength, well matched with natural soft clay

**Table 2:** Basic Properties of Raw Materials.

Material	Density 20 °C (g·cm^−3^)	Flash Point (°C)	Refractive Index	Kinematic Viscosity 40 °C (mm^2^·s^−1^)
Nano-fumed silica	0.078	-	1.4600	-
N-dodecane	0.824	66.5	1.4345	1.487
#15 white mineral oil	0.854	158	1.4625	16.25
Fused quartz sand	1.458	-	1.4585	-

**Table 3:** Mass proportions of materials for the formulation of transparent soft clay.

No.	Pore Fluid: n-Dodecane and White Oil *m*_1_ (g)			Solid Particle *m*_2_ + *m*_3_ (g)			Pore Fluid: Solid Particles *m*_1_/(*m*_2_ + *m*_3_) (%)
	n-Dodecane/#15 White Mineral Oil	Refractive Index	Mass of Pore Fluid *m*_1_ (g)	Fused Quartz Sand		Nano-Fumed Silica *m*_3_ (g)	
				Mass *m*_2_ (g)	Grade		
1	1:5	1.4576	1417	1000	(A)	181	120
2	1:5.2	1.4580	1416	1000	(A)	180	120
3	1:5.6	1.4584	1419	1000	(A)	182.5	120
4	1:6.2	1.4588	1416	1000	(A)	180	120
5	1:8	1.4594	1416	1000	(A)	180	120
6	1:10.4	1.4600	1418	1000	(A)	182	120
7	1:12	1.4602	1415	1000	(A)	179	120
8	1:6.2	1.4588	1416	1000	(A)	290	110
9	1:6.2	1.4588	1416	1000	(A)	90	130
10	1:6.2	1.4588	1416	1000	(B)	90	130
11	1:6.2	1.4588	1416	1000	(C)	90	130
12	1:6.2	1.4588	1416	1000	(D)	90	130
13	1:6.2	1.4588	1416	1000	(E)	90	130

Note: A–E represent five different aggregate gradations. Detailed particle size proportions of each gradation are shown in Figure 2b.

**Table 4:** Test Results of Density and Oil Content for Transparent Soft Clay.

No.	Refractive Index	$m_{1}$ (g)	$m_{2}$ (g)	$m_{3}$ (g)	*w* (%)	*ρ* (g·cm^−3^)
1	1.4584	11.23	21.68	15.81	128	1.26
2	1.4588	11.20	21.37	15.64	129	1.23
3	1.4592	11.59	21.47	15.92	128	1.21
4	1.4596	11.25	21.52	15.77	127	1.25

**Table 5:** Comparison of compression characteristic indices between transparent soft clay and regional soft clays.

Soil Layer Name	*ρ* (g·cm^−3^)	*w* (%)	$e_{0}$	$\alpha_{v(0.1-0.2)}$ (MPa^−1^)	$C_{c}$	$E_{s(0.1-0.2)}$ (MPa)	$C_{s}$
Transparent Soft Clay (This study)	1.26	127.9	1.454	1.43	0.478	1.72	0.034
Shanghai No.4 Gray Silty Clay	1.73	50.0	1.361	1.02	0.541	2.23	−
Wenzhou, Zhejiang Undisturbed Silty Clay	1.61	62.8	1.776	1.16	0.396	2.39	−
Lianyungang Undisturbed Silty Clay	−	66.4	1.775	2.20	0.664	1.26	−
Kitakyushu clays	−	59.4	1.582	1.60	0.548	1.61	−
New York clays	−	87.0	2.341	2.51	0.741	1.33	−

**Table 6:** Test Scheme.

Test Name	Sample Name	Model Pile Diameter (mm)	Model Pile Length (mm)	Loading Rate (mm·s^−1^)
Jacked-Pile Penetration Model Test	Condition 1 (A1, A2)	16	300	0.067
	Condition 2 (A3, A4)	20		
	Condition 3 (A5, A6)	24		

**Table 7:** Tip resistance, shaft friction, and their respective proportions in total jacked-pile resistance upon the completion of pile installation.

Test Pile	Pile Driving Resistance (N)	Tip Resistance (N)	Percentage of Tip Resistance (%)	Shaft Friction (N)	Percentage of Shaft Friction (%)
Condition 1	8.99	0.79	9%	8.20	91%
Condition 2	12.35	1.78	14%	10.57	86%
Condition 3	16.08	3.18	20%	12.66	79%

## Data Availability

The original contributions presented in this study are included in the article. Further inquiries can be directed to the corresponding author.

## List of Notations

*n*	Refractive index of pore liquid
*P*	Mass fraction of fluid
*w*	Oil content of transparent soil
$\alpha_{v}$	Compression coefficient
$e_{0}$	Pore ratio
$C_{c}$	Compression Index
$E_{s}$	Compression modulus
$C_{s}$	Rebound Index
*c*	Cohesion
*φ*	Internal friction angle
*m*_1_	Mass of the pore–fluid mixture
*m*_2_	Mass of the fused-quartz sand particles
*m*_3_	Mass of the nano-fumed silica powder
$n_{12}$	Refractive index of mixed pore fluid
$n_{1}$	Refractive index of individual fluid 1
$n_{2}$	Refractive index of individual fluid 2
$P_{1}$	Component mass fraction of fluid 1
$P_{2}$	Component mass fraction of fluid 2
